# Challenges of EGFR-TKIs in NSCLC and the potential role of herbs and active compounds: From mechanism to clinical practice

**DOI:** 10.3389/fphar.2023.1090500

**Published:** 2023-04-07

**Authors:** Xiaotong Song, Luchang Cao, Baoyi Ni, Jia Wang, Xiaoyan Qin, Xiaoyue Sun, Bowen Xu, Xinmiao Wang, Jie Li

**Affiliations:** ^1^ Department of Oncology, Guang’ Anmen Hospital, China Academy of Chinese Medical Sciences, Beijing, China; ^2^ Department of Respiratory, Hongqi Hospital Affiliated to Mudanjiang Medical College, Mudanjiang, China; ^3^ Institute of Acupuncture and Moxibustion, China Academy of Chinese Medical Sciences, Beijing, China

**Keywords:** EGFR-TKIs, non-small cell lung cancer, resistance, adverse events, herbs, active compounds

## Abstract

Epidermal growth factor receptor (EGFR) mutations are the most common oncogenic driver in non-small cell lung cancer (NSCLC). Epidermal growth factor receptor-tyrosine kinase inhibitors (EGFR-TKIs) are widely used in the treatment of lung cancer, especially in the first-line treatment of advanced NSCLC, and EGFR-TKIs monotherapy has achieved better efficacy and tolerability compared with standard chemotherapy. However, acquired resistance to EGFR-TKIs and associated adverse events pose a significant obstacle to targeted lung cancer therapy. Therefore, there is an urgent need to seek effective interventions to overcome these limitations. Natural medicines have shown potential therapeutic advantages in reversing acquired resistance to EGFR-TKIs and reducing adverse events, bringing new options and directions for EGFR-TKIs combination therapy. In this paper, we systematically demonstrated the resistance mechanism of EGFR-TKIs, the clinical strategy of each generation of EGFR-TKIs in the synergistic treatment of NSCLC, the treatment-related adverse events of EGFR-TKIs, and the potential role of traditional Chinese medicine in overcoming the resistance and adverse reactions of EGFR-TKIs. Herbs and active compounds have the potential to act synergistically through multiple pathways and multiple mechanisms of overall regulation, combined with targeted therapy, and are expected to be an innovative model for NSCLC treatment.

## 1 Introduction

Lung cancer is a major killer of human life and health and the leading cause of cancer death worldwide, it has a poor prognosis with an overall 5-year survival rate of 20.5%. Global Cancer Statistics 2020 data shows that lung cancer ranks second in new cancer cases worldwide, accounting for 11.4% of cancer diagnoses and 18.0% of all cancer deaths (Sung et al., 2021). Smoking (Alberg and Samet, 2003), emissions of chemical fuels such as coal and petroleum, kitchen fumes, air pollution (Turner et al., 2020), secondhand smoke, advanced age (Force et al., 2021), history of pulmonary fibrosis, human immunodeficiency virus infection, and alcohol use (Hubbard et al., 2000; Kirk et al., 2007) have all been identified as risk factors for the development of lung cancer.

Lung cancer is divided into non-small cell lung cancer (NSCLC, accounting for about 85% of the total diagnosis) and small cell lung cancer (SCLC, accounting for about 15% of the total diagnosis). As the main type of lung cancer, NSCLC has a poor prognosis, especially in advanced NSCLC patients. Over the past 20 years, with the progress of precise treatment at the medical biomolecular level, targeted drug therapy has greatly revolutionized the diagnosis and treatment mode of lung cancer and gradually improved the prognosis of NSCLC patients (Thai et al., 2021). The 5-year survival rate for NSCLC in 2021 has reached 26.4% (vs. 23.2% in 2014) (Ganti et al., 2021). The frequencies of common oncogenic driver mutations in NSCLC are EGFR (17%), KRAS-non-G12C (17%), KRAS-G12C (12%), BRAF (5%), MET (4%), ERBB2 (4%), ALK (3%), RET (2%), ROS1 (1%), NTRK1/2/3 (<1%), and Other or not identified (32%) (Thai et al., 2021). EGFR is one of the major driver genes in NSCLC, and approximately 10% of American NSCLC patients and 35% of Eastern Asian NSCLC patients carry tumor-associated mutations in the EGFR gene (Lynch et al., 2004; Paez et al., 2004; Pao et al., 2004). In addition, the frequency of EGFR mutations was higher in women, non-smokers or light smokers, up to 59.4% (Li et al., 2016). The hotspot mutations of EGFR are mainly located in four exons of its kinase region: 18, 19, 20, and 21, and about 90% of the hotspot mutations are deletions in exon 19 and point mutations in exon 21 L858R (Jackman et al., 2009; Rosell et al., 2012).

EGFR is a member of the receptor tyrosine kinase (RTK) superfamily, consisting of exon boundaries and associated extracellular, transmembrane, and intracellular protein structural domains (Shi et al., 2022). Transmembrane glycoproteins include a cysteine-rich extracellular ligand-binding domain, a hydrophobic transmembrane domain, a cytoplasmic RTK domain, and a C-terminal domain. The RTK structure contained an N-flap consisting of five β-folded chains and an αC helix and a C-flap containing a highly flexible activation loop (A-loop) of the main helix (Amelia et al., 2022). This allows the EGFR receptor to bind to ligands helping cells receive signals and respond to their environment. EGFR binding to ligands promotes dimer accumulation and autophosphorylation, initiates cellular signaling cascades, and regulates cell proliferation and signal transduction. It activates downstream pathways such as RAS-RAF-MEK-ERK and PI3K-AKT-mTOR (Jorissen et al., 2003; Dawson et al., 2005). The RAS-RAF-MEK-ERK pathway is responsible for controlling gene transcriptional activity and cell cycle, whereas the PI3K-AKT-mTOR pathway activates anti-apoptotic signals. Tyrosine kinase inhibitors (TKIs) are naturally reversible or irreversible small molecules. They exist as adenosine triphosphate (ATP) analogs and inhibit EGFR signaling by competing with the ATP binding pocket on the intracellular catalytic kinase structural domain of the RTK, thereby preventing autophosphorylation and activation of several downstream signaling pathways (Ciardiello, 2000; Seshacharyulu et al., 2012). EGFR-TKIs have opened the era of precise treatment. While achieving clinical efficacy, the drawbacks of drug resistance have gradually emerged. How to further improve the anti-tumor activity of patients with EGFR mutations is currently a key issue. Scholars have been actively developing a new generation of targeted therapies for EGFR mutations, and the fourth generation of EGFR-Tkis is currently in clinical trials, such as BLU-945. In addition, the synergistic application of EGFR-TKIs with other therapies has also been found to have a certain anti-drug resistance potential.

## 2 EGFR-TKIs in synergistic treatment of NSCLC

Targeted EGFR-TKIs therapy has demonstrated its efficacy advantages in prolonging survival duration and inhibiting tumor growth after more than 20 years of exploratory clinical studies. And it is more selective and inhibitory with the upgrading of drugs (Tian et al., 2022). Although EGFR-TKIs targeted drugs have achieved efficacy progress compared with standard therapies, it is still necessary to pursue higher benefits for patients in clinical practice. Therefore, scholars began to explore whether EGFR-TKIs combined with other therapies can further improve the comprehensive efficacy. For example, a SINDAS trial (Wang et al., 2022) evaluating first-generation TKIs with or without radiotherapy for EGFR-mutated synchronous oligometastatic NSCLC. It randomized 133 NSCLC patients with synchronous oligometastases without brain metastases to TKIs alone or prior radiotherapy before TKIs. The results showed that median progression-free survival (PFS) was 12.5 months vs. 20.2 months (*p* < 0 .001) and median overall survival (OS) was 17.4 months vs. 25.5 months (*p* < 0.001) for TKIs treatment alone vs TKIs combined with radiotherapy. Therefore, this review focuses on summarizing the clinical studies of EGFR-TKIs combined therapy for NSCLC, which are elaborated according to different generations (Table 1).

**TABLE 1:** Clinical study of EGFR-TKIs in synergistic treatment of NSCLC.

Scheme	N	ORR (%)	PFS (mth)	OS (mth)	Toxicity
Gefitinib + Pemetrexed + Carboplatin vs. Gefitinib Blackhall et al. (2006)	350	75.3% vs. 62.5% (*p* = 0.01)	16 vs. 8 (HR 0.51, *p* < 0.001)	NR^a^ vs. 17 (HR 0.45, *p* < 0.001)	75% vs. 49.4% (*p* < 0.001)
Erlotinib + Bevacizumab vs. Erlotinib Noronha et al. (2020)	228	NR	28.6 vs. 24.3 (HR 0.773, *p* > 0.05)	50.7 vs. 46.2 (HR 1.007, *p* = 0.97)	NR
Erlotinib + Ramucirumab vs. Erlotinib + Placebo Nakagawa et al. (2019)	449	NR	19.4 vs. 12.4 (HR 0.59, *p* < 0.0001)	NR	29% VS. 21% (No *p*-value reported)
PP or DP chemotherapy + Icotinib vs. Icotinib Xing et al. (2021)	68	44.1% vs. 54.5%	13.4 vs. 8.0 (*p* = 0.0249)	36.0 vs. 23.1 (*p* = 0.4511)	NR
Afatinib + Cetuximab vs. Afatinib Goldberg et al. (2020)	168	74% vs. 67% (*p* = 0.38)	13.4 vs. 11.9 (HR 1.01, *p* = 0.94)	67% vs. 70% (2-year OS, %) (HR 0.82, *p* = 0.44)	72% vs. 40% (*p* < 0.0001)
Osimertinib + Bevacizumab vs. Osimertinib Soo et al. (2022)	155	55% vs. 55% (*p* = 0.17)	15.4 vs. 12.3 (HR 0.96, *p* = 0.83)	24.0 vs. 24.3 (HR 1.03, *p* = 0.91)	96.1% vs. 87.0% (No *p*-value reported)

^a^ NR, not reached.

### 2.1 First generation EGFR-TKIs

#### 2.1.1 Gefitinib

Gefitinib is the world’s first specific molecular targeted agent developed for the treatment of NSCLC, which was approved by the US Food and Drug Administration (FDA) in 2003 for the treatment of advanced chemo-refractory NSCLC (Blackhall et al., 2006). Gefitinib binds competitively to the ATP-binding pocket of EGFR in a reversible manner, thereby inhibiting autophosphorylation. However, the clinical efficiency of gefitinib depends on EGFR subtype: patients with exon 19 deletions or L858R mutations benefit more than EGFR wild-type patients (Paez et al., 2004). Gefitinib binds closer to the hinge region of L858R mutant EGFR with high affinity for mutant EGFR compared to wild-type EGFR (Liu et al., 2006). Expression of hyperdifferentiation/DNA binding inhibitor 1 (ID1) in NSCLC activates epithelial mesenchymal transition in NSCLC cells to promote the formation of pre-metastatic ecological sites in the liver and promote metastasis (Kim et al., 2013; Castañón et al., 2017). Upon gefitinib intervention, ID1 overexpression in NSCLC induces necroptosis through RIP3/MLKL upregulation and cFLIPS-induced RIP1 dissociation (Tan et al., 2020).

A Phase III randomized trial (Mitsudomi et al., 2010) investigated the prognostic difference between gefitinib and gefitinib + pemetrexed + carboplatin chemotherapy in EGFR-mutated NSCLC. 350 patients with EGFR-mutated advanced NSCLC scheduled for first-line palliative therapy were randomly assigned to gefitinib group (*N* = 176) and gefitinib + chemotherapy group (*N* = 174) with a median follow-up time of 17 months. The results showed that the median PFS of gefitinib + chemotherapy group reached 16 months (95% CI, 13.5–18.5 months), the median OS was significantly longer than that of gefitinib group [not reached vs. 17 months (95% CI, 13.5–20.5 months)]. It can be seen that gefitinib combined with chemotherapy showed a prognostic advantage in PFS and OS compared with gefitinib alone, but both groups showed clinical grade 3 or higher toxicity, and the benefits and risks were difficult to balance.

#### 2.1.2 Erlotinib

Erlotinib selectively occupies ATP binding sites and reverses inhibition of EGFR autophosphorylation similar to gefitinib. Erlotinib-mediated interaction of BECN1 with autophagy inhibitory proteins RUBCN/Rubicon and BCL2 induces autophagy (Cao et al., 2020).

A global, double-blind, phase III clinical trial (Nakagawa et al., 2019) evaluated the survival of ramucirumab + erlotinib versus placebo + erlotinib in treatment-naïve metastatic NSCLC patients with EGFR mutations. 449 NSCLC patients with no CNS metastases randomized to oral erlotinib + intravenous ramucirumab or placebo. The results showed that PFS of 19.4 months (95% CI, 15.4–21.6) was significantly longer in the combined treatment group than 12.4 months (95% CI, 11.0–13.5) in the placebo group. Ramucirumab + erlotinib showed a longer PFS compared with placebo + erlotinib. Another phase III, multicenter, randomized trial (Stinchcombe et al., 2019; Kawashima et al., 2022) observed the OS analysis of bevacizumab + erlotinib versus erlotinib alone in Japanese patients with advanced metastatic EGFR-mutated NSCLC (NEJ026). Included a total of 228 patients who were randomly divided into oral erlotinib + bevacizumab or erlotinib alone. The results showed that the median OS was 50.7 months and the median PFS was 28.6 months in the bevacizumab + erlotinib group. Median OS 46.2 months and median PFS 24.3 months in the erlotinib alone group. Erlotinib + bevacizumab did not prolong survival compared with erlotinib alone, it may be affected by multiple factors such as treatment, region, and population. In addition, a multicenter, randomized, open-label, phase II trial (Xing et al., 2021) compared the efficacy of erlotinib or etoposide and cisplatin (EP) chemotherapy versus concurrent radiotherapy (RT) in patients with stage IIIA/B unresectable advanced NSCLC with EGFR mutations. 252 patients were randomized to Erlotinib + RT or EP + RT. The results showed that erlotinib + RT prolonged PFS better than EP + RT (24.5 m vs. 9.0 m, *p* < 0.01). Median OS, overall response rate (ORR), and incidence of adverse events showed no significant statistical difference.

#### 2.1.3 Icotinib

A multicenter, randomized controlled trial (Sun et al., 2022) was conducted to evaluate the efficacy and safety of icotinib versus first-line chemotherapy followed by sequential icotinib maintenance therapy in the treatment of advanced EGFR mutant NSCLC. 68 patients with EGFR mutant stage IIIB/IV NSCLC were randomly divided into icotinib group and first-line chemotherapy (PP or DP regimen) followed by icotinib group. The results showed that the median PFS was 8.0 m vs. 13.4 m (*p* < 0.05), and the median OS was 23.1 m vs. 36.0 m (*p* > 0.05) in icotinib alone group and chemotherapy followed by icotinib alone group, respectively. Compared with icotinib alone group, sequential chemotherapy could prolong PFS. Therefore, sequential chemotherapy may be a potential treatment strategy for NSCLC patients with EGFR mutation.

### 2.2 Second generation EGFR-TKIs

The second-generation EGFR-TKIs has the same quinazoline scaffold as the first-generation EGFR-TKIs, but the side chain can irreversibly bind to Cys797 and inhibit the tyrosine kinase activity of EGFR. For example, anilinyl quinazoline derivatives form hydrogen bonds with the backbone of Met793 in the hinge region and interact with the hydrophobic region (Shi et al., 2022a).

#### 2.2.1 Afatinib

Afatinib, a second-generation EGFR-TKIs with irreversible inhibition of pan-ErbB, was developed for NSCLC with EGFR mutations (Li et al., 2008). Afatinib downregulates ErbB signaling by binding to the kinase structural domains of EGFR, ErbB2 and ErbB4 and irreversibly inhibiting tyrosine kinase autophosphorylation (Laskin et al., 2020). In two preclinical studies in which EFM-19 cells were exposed to a conditioned medium model of human lung cancer cells expressing an exogenous CD74-NRG1 fusion protein and a xenograft model of NRG1 rearrangement patient origin, afatinib was shown to have antitumor activity and downstream ErbB signaling inhibition (Nakaoku et al., 2014; Drilon et al., 2018). Early in treatment, afatinib can inhibit CD8^+^ T lymphocyte proliferation by targeting CAD, providing a time window for combination therapy to prevent EGFR-TKIs from inhibiting ICB efficacy (Tu et al., 2021). For lung cancer patients with rare EGFR mutations in the tumor, such as EGFR L858M/L861Q cis mutation (Saxon et al., 2017), EGFR S768I, L861Q, G719X, and S768I mutations, afatinib is considered to be effective (Wu et al., 2014; Yang et al., 2015).

A phase II, multicenter trial (Goldberg et al., 2020) conducted to investigate whether afatinib + cetuximab improves the prognosis of patients with EGFR-mutated NSCLC compared with afatinib alone. 168 treatment-naive patients were included with advanced EGFR-mutated NSCLC who were randomized to afatinib + cetuximab or afatinib alone. The results showed that PFS was not significantly improved in afatinib + cetuximab (13.4 vs. 11.9 m, *p* > 0.05), compared to afatinib alone. But the incidence of adverse events in conbination was increased (72% vs. 40%). Previous studies (Ninomiya et al., 2013) have found that afatinib combined with bevacizumab showed synergistic effect in TKI-resistant xenograft lung cancer models, therefore, a multicenter, single-arm, phase 2 trial (Hata et al., 2018) was conducted to evaluate the clinical efficacy and safety of afatinib combined with bevacizumab in the treatment of acquired resistance (AR). 32 NSCLC patients after AR to EGFR-TKIs were enrolled and treated with afatinib and bevacizumab until progression. The results showed that the median PFS was 6.3 m, and subgroup analysis of the T790M + and T790M—patient populations showed median PFS times of 6.3 and 7.1 months, respectively. Suggesting that afatinib combined with bevacizumab has certain clinical efficacy and safety after AR to EGFR TKIs and may be a therapeutic optimization option for the T790M population.

#### 2.2.2 Dacomitinib

Daclatinib is a second-generation irreversible inhibitor of EGFR and pan-HER. Daclatinib binds to ErbB family members in the ATP binding domain and covalently modifies nucleophilic cysteine residues in an irreversible manner (Engelman et al., 2007; Gonzales et al., 2008). The semi-inhibitory concentration of daclatinib on ERBB2 and ERBB4 is more than 6 times lower than that of gefitinib and erlotinib (Rawluk and Waller, 2018). In phase III clinical trials for the treatment of NSCLC, daclatinib induced protective autophagy to reduce its anti-cancer effects. Its combination with cepharanthine increased the anti-proliferative and apoptotic effects of DAC *in vitro* and enhanced the anti-cancer effects of DAC in NCI-H1975 xenograft mice (Tang et al., 2018). Daclatinib with the estrogen antagonist fulvestrant downregulated activator protein 1 and reversed the prognosis-associated gene signature of c-Myc, MIA, CXXC5, FGFR4, FOXC1, and Grb in NSCLC (Almotlak et al., 2020).

Previous studies have demonstrated that combined targeting of EGFR and MEK is more effective than single agents in treating cancer with EGFR T790M and can hinder the development of acquired resistance in mutant EGFR lung cancer (Tricker et al., 2015). EGFR T790M mutation is present in approximately 50%–60% of patients and is the most common mechanism of acquired resistance to first- and second-generation EGFR-TKIs (Westover et al., 2018). Therefore, some scholars have proposed the idea that the combination of dacitinib and osimertinib may be an effective first-line treatment for patients with advanced EGFR, and proposed a variety of computational strategies. A predictive modeling platform was developed to determine the optimal dosing regimen for the targeted therapy combination, resulting in a combination strategy of oxitinib + dasitinib that is expected to minimize tumor burden with tolerable adverse effects. This phase I clinical trial (NCT03810807) is ongoing (Yu, 2019).

### 2.3 Third generation EGFR-TKIs

Third-generation covalent inhibitors are irreversibly bound to the target and are mutation selective. These compounds were designed based on a novel aminopyrimidine scaffold and showed better biological activity (Pao and Chmielecki, 2010).

#### 2.3.1 Osimertinib

Clinical studies have shown significant positive results of osimertinib as first-line treatment for untreated advanced NSCLC with EGFR-activating mutations, with superior efficacy to standard EGFR-targeted therapy (Ramalingam et al., 2018; Soria et al., 2018). Osimertinib is an FDA-approved drug for the first-line treatment of advanced NSCLC with EGFR-activating mutations. It has also been used as second-line therapy for patients with EGFR T790M-mutated NSCLC that has relapsed from a first-generation EGFR-TKI (Zhang et al., 2021).

Death receptor 4 (DR4) expression is a poor prognostic factor in human lung adenocarcinoma. Treatment with osimertinib effectively and rapidly reduced DR4 levels in PC-9 and HCC827 (Zhang et al., 2021). The reason for this is that osimertinib inhibits MEK/ERK signaling in EGFR-sensitive NSCLC cells (Yao et al., 2016). One of the hallmarks of cancer is the reprogramming of lipid metabolism. Cancer cells exhibit significant metabolic alterations to support cell proliferation. Osimertinib promotes degradation of the mature form of sterol regulatory element binding protein (SREBP1) in a GSK3/FBXW7-dependent manner and reduces its protein levels of regulatory genes in EGFR mutant NSCLC cells/tumors, while inhibiting adipogenesis (Chen et al., 2021b).

A randomised phase II study of bevacizumab (BOOSTER) (Soo et al., 2022) was conducted to investigate the efficacy and safety of Osimertinib combined with bevacizumab compared to osimertinib alone, with a median follow-up time of 33.8 months. It included 155 patients with advanced NSCLC with EGFR T790M mutation after failure of previous EGFR TKI therapy. It was found that there was no statistical difference in PFS or OS between osimertinib + bevacizumab and osimertinib alone (PFS: 15.4 m vs. 12.3 m, OS: 24.0 m vs. 24.3 m, *p* > 0.05 for all), while grade ≥3 AEs were more common when combined (47% vs. 18%). Another open-label, randomised phase 2 clinical trial (Tanaka et al., 2021) first compared the efficacy and tolerability of osimertinib combined with carboplatin + pemetrexed with osimertinib monotherapy in EGFR mutation-positive NSCLC patients. 62 patients were included who were randomly divided into osimertinib monotherapy group or combination group. The results showed that osimertinib in combination with chemotherapy, although tolerated, did not prolong survival (the median PFS: 14.6 m vs. 15.8 m, *p* > 0.05, ORR: 53.6% vs. 71.4%).

#### 2.3.2 Aumolertinib

Aumolertinib (formerly almonertinib) is a well-tolerated third-generation epidermal growth factor receptor tyrosine kinase inhibitor that was recommended as first-line therapy for patients with EGFR-mutated NSCLC in 2022. Previous studies showed (Lu et al., 2022) that first-generation EGFR-TKIs combined with radiotherapy improved median PFS compared with conventional chemotherapy combined with radiotherapy (24.5 vs. 9.0 months, *p* < 0.01), revealing the potential clinical value of EGFR-TKIs combined with radiotherapy. And third-generation EGFR-TKIs have been found to increase radiosensitivity in EGFR-mutated NSCLC (Wu et al., 2018). A multicenter phase II clinical study (Zhu et al., 2021b) was carried out to explore the safety and efficacy of third-generation EGFR-TKIs combined with thoracic radiotherapy in locally advanced EGFR-mutated NSCLC. The patients were divided into group A treated with amolertinib followed by radiotherapy after 2 months and group B treated with amolertinib concurrent radiotherapy. The incidence of grade ≥3 radiation pneumonitis within 6 months after radiotherapy, and the secondary outcome measures include PFS and OS were observed. This study is ongoing (ClinicalTrials.gov NCT04636593).

#### 2.3.3 Lazertinib

Lazertinib, a mutant selective third-generation EGFR-TKIs that penetrates the blood-brain barrier, has superior therapeutic efficacy in EGFR-mutant brain metastasis models. Lazetinib showed good inhibition of metastatic brain tumors from lung cancer and demonstrated more selective, superior antitumor activity. It has a lower incidence of cutaneous adverse reactions compared to osimertinib (Yun et al., 2019). Lazertinib demonstrated tolerable safety in NSCLC and achieved preliminary efficacy in clinical trials (Ahn et al., 2019). A multicenter Two-arm, phase II trial (Kim et al., 2022) was conducted in Korea in 2021 to explore the clinical efficacy and safety of lazertinib combined with upfront local ablative radiotherapy in patients with EGFR-mutated NSCLC with synchronous oligometastatic disease (ClinicalTrials.gov NCT05167851). Patients were randomized to receive Lazertinib or Lazertinib + stereotactic body radiation therapy (SBRT), mainly observing PFS, OS, ORR, and other outcome measures, and the study is currently ongoing.

## 3 The challenges of EGFR-TKIs for NSCLC

### 3.1 Mechanisms of resistance to EGFR-TKIs

**FIGURE 1:**
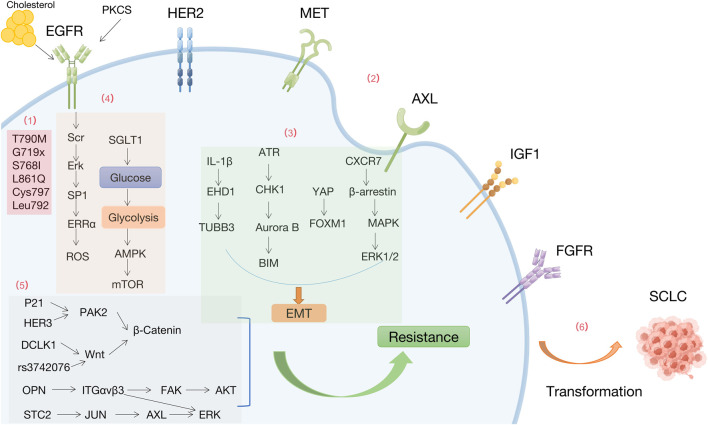
Mechanisms of resistance to EGFR-TKIs by Figdraw. (1) EGFR-dependent mutations (2) Bypass activation (3) Epithelial-mesenchymal transition (4) Metabolism. (5) Downstream signals (6) Histology and phenotypic transformation.

EGFR-TKIs represent a major advance in the treatment of NSCLC with EGFR-activating mutations. However, the development of drug resistance remains a major obstacle to the clinical efficacy of EGFR-TKIs. Disease progression occurs after approximately 9–12 months of targeted therapy with first- and second-generation EGFR-TKIs (Mitsudomi et al., 2010; Rosell et al., 2012) (Figure 1).

#### 3.1.1 EGFR-dependent mutations

Resistance to first-generation EGFR-TKIs occurs due to EGFR T790M mutations (substitution of Thr790 by methionine in the hydrophobic ATP binding site encoded on exon 20), subclonal selection (genetically resistant clones) and rare EGFR mutations (e.g., G719X, S768I, and L861Q). Mutations such as T790M can still occur after second-generation EGFR-TKI treatment, with limited selectivity for WT-EGFR, resulting in severe side effects. Osimertinib covalently interacts with conserved cysteine residues in EGFR (Cys797) (Zhang, 2016), while the EGFR-C797S mutation is the most common resistance mutation, detected in 7% of osimertinib-resistant patients. A study found that rapid acquisition of the T790M mutation in circulating tumor DNA was detected in an S768I mutation carrier after only 3 months of afatinib treatment and may account for the low activity of afatinib activity (Russo et al., 2017). Mutations in Leu792, including L792F, L792Y, and L792H, introduce the benzene or imidazole ring into the 792 sides chain of the residue, spatially disrupting the oxytetracycline orientation and affects the binding of osimertinib to the EGFR ATP binding site (Ou et al., 2017).

#### 3.1.2 Bypass activation

Activation of bypass receptors is another important factor contributing to resistance to EGFR-TKIs, including amplification or activation of cMet, her2, AXL, IGFR1, and FGFR.

Aberrant activation of the proto-oncogene MET leads to EGFR-TKIs resistance. Expression of histone methyltransferase EZH2 negatively correlates with MET activation and EGFR-TKIs resistance in NSCLC cells and clinical samples. The “MET-AKT-EZH2” feedback loop regulates EGFR-TKIs resistance (Tsang et al., 2015). Deletion of the epigenetic factor lysine methyltransferase 5C (KMT5C) promotes resistance to multiple EGFR inhibitors, including erlotinib, gefitinib, afatinib, and osimertinib, in NSCLC *via* LINC01510-mediated MET upregulation (LINC01510/MET axis) (Pal et al., 2022).

Fibroblast growth factor receptor (FGFR) is a transmembrane RTK. Osimertinib-resistant patients have amplified FGFR1 and elevated fibroblast growth factor 2 (FGF2) mRNA levels (Kim et al., 2015). Patients with T790M mutation showed disease progression after treatment with osimertinib and nilotinib. FGFR3-TACC3 fusion was detected in the ctDNA from this patient (Tanaka et al., 2019). Hypoxia is a critical microenvironmental stress in solid tumors and is associated with acquired resistance to conventional therapy in NSCLC. Hypoxia-induced EGFR-TKIs resistance is driven by overexpression of FGFR1 to maintain ERK signaling (Lu et al., 2020). These findings suggest that abnormalities in the FGFR signaling pathway may underlie the mechanism of acquired resistance to third-generation EGFR-TKIs.

Insulin-like growth factor receptor 1 (IGF1R) is a transmembrane heterotetrameric protein, encoded by a gene located on chromosome 15q26.3, involved in promoting tumor cell growth. Aberrant activation of IGF1R leads to EGFR-TKIs resistance (Shi et al., 2022a).

In the context of NSCLC carrying EGFR oncogenic mutations, elevated levels of AXL and GAS6 have been found to confer resistance to EGFR-TKIs (e.g., erlotinib and osimertinib) in certain tumors with mesenchymal-like features. Moreover, AXL expression is regulated through a stochastic mechanism centered on epigenetic regulation of miR-335 (Safaric Tepes et al., 2021).

MERTK, a member of the TAM (TYRO3, AXL and MERTK) family of RTKs, is overexpressed or ectopically expressed in approximately 70% of NSCLC and is an attractive biological target for the treatment of NSCLC (Linger et al., 2013; Graham et al., 2014). Significant upregulation of MERTK and/or its ligands in EGFRMT tumors after treatment with osimertinib in xenograft models and patient samples activates paracrine signaling (Yan et al., 2022a).

#### 3.1.3 Epithelial-mesenchymal transition (EMT)

NSCLC cells with acquired resistance to gefitinib or osimertinib exhibit EMT characteristics with reduced E-cadherin and increased expression of wave proteins and Hakai (Shi et al., 2022a). Osimertinib resistance by EMT activates the ATR-CHK1-Aurora B signaling cascade, resulting in a hypersensitive response to the respective kinase inhibitor by activating BIM-mediated mitogenesis (Amelia et al., 2022). Integrating transcriptomic, proteomic and drug screening approaches, the data reveal that the YAP/FOXM1 axis is a central regulator of EMT-associated EGFR-TKIs resistance (Jorissen et al., 2003). CXCR7 (an atypical G protein-coupled receptor) activates the MAPK-ERK pathway *via* β-arrestin. Depletion of CXCR7 inhibits the MAPK pathway, significantly attenuates EGFR-TKI resistance, and leads to EMT. while CXCR7 overexpression reactivates ERK1/2 to confer EGFR-TKI resistance (Becker et al., 2019).

#### 3.1.4 Metabolism

Prolonged exposure to EGFR-TKIs generates drug resistance characterized by cholesterol accumulation in lipid rafts, which promotes EGFR and Src interactions and leads to EGFR/Src/Erk signaling activation-mediated SP1 nuclear translocation and ERRα re-expression. Functionally, ERRα re-expression maintained cell proliferation by regulating the ROS detoxification process and promoted the survival of gefitinib and oseltinib-resistant cancer cells. Patients with NSCLC who smoke has a lower prognosis and survival rate than those without a history of smoking. Exposure to cigarette smoke extracts enhances glycolysis and attenuates AMP-activated protein kinase (AMPK)-dependent inhibition of mTOR (Pan et al., 2022). Reduced glucose uptake is associated with the antitumor activity of EGFR-TKIs. PKCδ/EGFR axis-dependent SGLT1 upregulation is a key mechanism for acquired resistance to EGFR-TKI, and increased glucose uptake in NSCLC cells (Chen et al., 2021a).

#### 3.1.5 Exosomes

Intercellular transfer of exosomal wild-type EGFR promotes osimertinib resistance in NSCLC. Osimertinib promotes exosome release through upregulation of Rab GTPase (RAB17). Exosomes can be internalized by EGFR-mutated cancer cells through lattice-protein-dependent endocytosis, and then encapsulated exosomal wild-type EGFR protein then activates the downstream PI3K/AKT and MAPK signaling pathways and triggers osimertinib resistance (Wu et al., 2021).

#### 3.1.6 Immune evasion

HGF, MET-amplification and EGFR-T790M upregulate PD-L1 expression in NSCLC through different mechanisms, attenuating lymphocyte activation and cytotoxicity *in vitro* and *in vivo*, and promoting immune escape of tumor cells (Peng et al., 2019).

#### 3.1.7 Downstream signals

Significantly higher c-Myc levels in different EGFRm NSCLC cell lines with acquired resistance to osimertinib and in patients with relapsed EGFR-TKIs treatment compared to their corresponding parental cell lines (Zhu et al., 2021a). Phosphoproteomic analysis using global mass spectrometry in osimertinib-resistant NSCLC patients identified the activated p21-activated kinase 2 (PAK2)/β-catenin axis as a driver of osimertinib resistance. HER3, an upstream regulator of PAK2, drives activation of the PAK2/β-catenin pathway in osimertinib-resistant cells (Yi et al., 2022). Tumor stem cell marker DCLK1 is essential for maintaining tumor cell stemness *via* the Wnt/β-Catenin pathway and facilitates the development of EGFR-TKIs resistance (Yan et al., 2022b). FOXM1 variant rs3742076 leads to gefitinib resistance by activating the Wnt/β-Catenin signaling pathway in NSCLC patients (Guan et al., 2022).

HER2D16 can form homodimers in NSCLC cells. H1975 cells HER2D16-expressing H1975 cells are resistant to osimertinib treatment. HER2D16 can lead to osimertinib resistance through an Src-independent pathway (Hsu et al., 2020). OPN is overexpressed in acquired EGFR-TKIs-resistant NSCLC. OPN promotes acquired EGFR-TKIs resistance by upregulating integrin αVβ3 expression, which activates the downstream FAK/AKT and ERK signaling pathways to promote NSCLC cell proliferation (Fu et al., 2020). STC2 overexpression in EGFR-TKIs-sensitive cells leads to EGFR-TKIs resistance, and STC2 enhances AXL promoter activity by increasing phosphorylation of c-Jun. Therefore, STC2-JUN-AXL-ERK signaling may be a potential therapeutic target to overcome resistance to EGFR-TKIs (Liu et al., 2019).

Alternative pathways of aberrant activation, such as excessive STAT3 activation, may also lead to acquired EGFR-TKI resistance (Wheeler et al., 2010; Zulkifli et al., 2017). Patients with EGFR-TKIs-acquired resistant cancers have high levels of phosphorylated STAT3 to some extent (Hirsch et al., 2017; Herbst et al., 2018). Hsp90 inhibitors that block the N-terminal ATP binding pocket lead to transcriptional upregulation of Wnt ligands *via* Akt- and ERK-mediated STAT3 activation. Upregulation, which leads to the survival of NSCLC cells in an autocrine or paracrine manner (Lee et al., 2022).

#### 3.1.8 Histology and phenotypic transformation

3%–15% of NSCLC patients convert to SCLC histopathology and therefore develop acquired resistance to EGFR-TKIs (Norkowski et al., 2013; Dorantes-Heredia et al., 2016). This conversion occurs mainly in non-smokers with EGFR-TKIs-sensitive mutations (e.g., EGFR ex19del/T790M mutation) in Asian adenocarcinoma patients. RB1 and TP53 mutations, PI3K/AKT family activation and NOTCH signaling downregulation, MYC and SOX families, and AKT pathway activation are all involved in SCLC transformation (Yin et al., 2022). In addition, squamous cell transformation occurred in approximately 15% of patients receiving osimertinib as first- and second-line therapy, identified as another mechanism of acquired EGFR-TKIs resistance (Schoenfeld et al., 2020).

### 3.2 EGFR-TKIs-associated adverse events

EGFR-TKIs have become the first-line drugs for patients with EGFR mutation-positive advanced NSCLC. Although EGFR-TKIs provide longer PFS and better quality of life for patients with NSCLC, the occurrence of adverse events seriously affects patients’ continued use of this drug. The toxicity cannot be ignored. Adverse events are common with EGFR-TKIs drugs, such as digestive reactions (diarrhea and liver damage) and skin reactions (rash and onychomycosis, etc.).

#### 3.2.1 EGFR-TKIs-associated gastrointestinal adverse events

Diarrhea occurs more frequently after treatment with EGFR-TKIs, but the majority of people have mild symptoms, such as loose stools, watery stools, mucopurulent stools or pus and blood, and in severe cases, dehydration. In a clinical study that included 2,535 patients treated with EGFR-TKIs, 53.3% developed diarrhea (Ding et al., 2017). The probability of EGFR-TKIs causing diarrhea was: afatinib (78%, *n* = 223) (Cappuzzo et al., 2015), nazartinib (47%, *n* = 45) (Tan et al., 2022), limertinib (81.7%, *n* = 289) (Shi et al., 2022b), osimertinib (58%, *n* = 278) (Soria et al., 2018), and abivertinib (75%, *n* = 52) (Ma et al., 2018), naquotinib (47%, *n* = 110) (Yu et al., 2017), and mobocertinib (83%, *n* = 136) (Riely et al., 2021).

Liver injury can occur within 7 days-6 months after TKIs treatment, with non-specific symptoms such as malaise, loss of appetite, distension in the liver area and gastrointestinal symptoms. After drug administration, liver function should be tested regularly and liver protection, glucocorticoids and nutritional support should be performed in case of abnormalities.

Hepatic dysfunction is a prominent dose-limiting toxicity of Gefitinib. Gefitinib can selectively degrade cytochrome c oxidase subunit 6A1 (COX6A1)-an important anti-apoptotic factor in the autophagy-lysosomal pathway. Gefitinib-induced COX6A1 reduction impairs the function of mitochondrial respiratory chain complex IV (RCC IV), which in turn activates apoptosis, resulting in liver damage (Luo et al., 2021).

#### 3.2.2 EGFR-TKIs-related skin adverse events

Skin adverse events caused by EGFR-TKIs include rash/acne-like rash, dry skin, itching, scalp damage (alopecia folliculitis, hirsutism), and inflammation of nails/periungual tissues. Rash/acne-like rash and paronychia are the most common adverse event.

EGFR-TKIs-induced rash/acne-like rash mostly occurs 1 week-2 weeks after targeted drug therapy. The rash mostly occurs in areas rich in sebaceous glands and in severe cases can involve the lower extremities or even the whole body, affecting the patient’s quality of life. The probability of rash in EGFR-TKIs was: icotinib (14.8%, *n* = 418) (Shi et al., 2017), dacomitinib (14%, *n* = 227) (Wu et al., 2017), afatinib (83%, *n* = 223) (Cappuzzo et al., 2015) nazartinib (38%, *n* = 45) (Tan et al., 2022), and limertinib (29.9%, *n* = 289) (Shi et al., 2022b), poziotinib (48.9%, *n* = 90) (Le et al., 2022), osimertinib (32%, *n* = 279) (Tanaka et al., 2021), lazertinib (30%, *n* = 127) (Ahn et al., 2019), mobocertinib (33%, *n* = 136) (Riely et al., 2021).

Paronychia mostly appears 4–8 weeks after initial treatment and can occur in any nail or toenail, mainly as redness, swelling, pain and, in severe cases, inflammation, ulceration and purulent granulation tissue. In addition to the common rash and paronychia, there are a number of rare cutaneous adverse events caused by EGFR-TKIs, such as toxic epidermal necrolysis relaxans (Sheen et al., 2020).

#### 3.2.3 EGFR-TKIs-associated oral mucositis

Oral mucositis mostly occurs with second-generation EGFR-TKIs (afatinib, dacomitinib, etc.), often appearing on the 13th-19th day of drug initiation, with an incidence of generally about 15%. The probability of stomatitis with EGFR-TKIs is poziotinib (24.4%, *n* = 90) (Le et al., 2022) and nazartinib (27%, *n* = 45) (Tan et al., 2022). The vast majority of symptoms were mild and grade 3 or higher not being common. Patients develop erythema, edema, and erosion of the oral mucosa, further forming punctate and flaky ulcers, eventually causing pain, dysphagia, and abnormal taste.

#### 3.2.4 EGFR-TKIs-associated interstitial lung disease

Interstitial lung disease (ILD) is a rare and fatal adverse event induced by EGFR-TKIs (Tsubata et al., 2022). It is a lesion characterized by non-infectious inflammatory changes and progressive fibrosis in the focal or diffuse interstitial lung stroma, even developing into respiratory failure and cardiac insufficiency. It usually occurs within 3–7 weeks after treatment with EGFR-TKIs. EGFR-TKIs-induced ILD is low at 1.1%–2.2%, but accounts for 58% of all EGFR-TKI treatment-related deaths (Ohmori et al., 2021).

#### 3.2.5 EGFR-TKIs-associated cardiotoxicity

Relative to other EGFR inhibitors and other targeted therapies, osimertinib has a strong cardiotoxicity signal. There are case reports of cardiotoxicity due to osimertinib in combination with other drugs and death due to treatment failure (Bian et al., 2020). Osimertinib is strongly associated with QT prolongation, SVT and heart failure. Of the 4,095 ADRs with osimertinib, there were 179 cases of heart failure (3.7%) and 143 cases of arrhythmias (2.9%) (Waliany et al., 2021). Osimertinib was associated with a 2.2-fold higher rate of heart failure, a 2.1-fold higher rate of atrial fibrillation, and a 6.6-fold higher rate of QT prolongation compared with other EGFR inhibitors for NSCLC (Anand et al., 2019).

## 4 The potential role of herbs and active compounds in overcoming drug resistance and adverse effects of EGFR-TKIs

**FIGURE 2:**
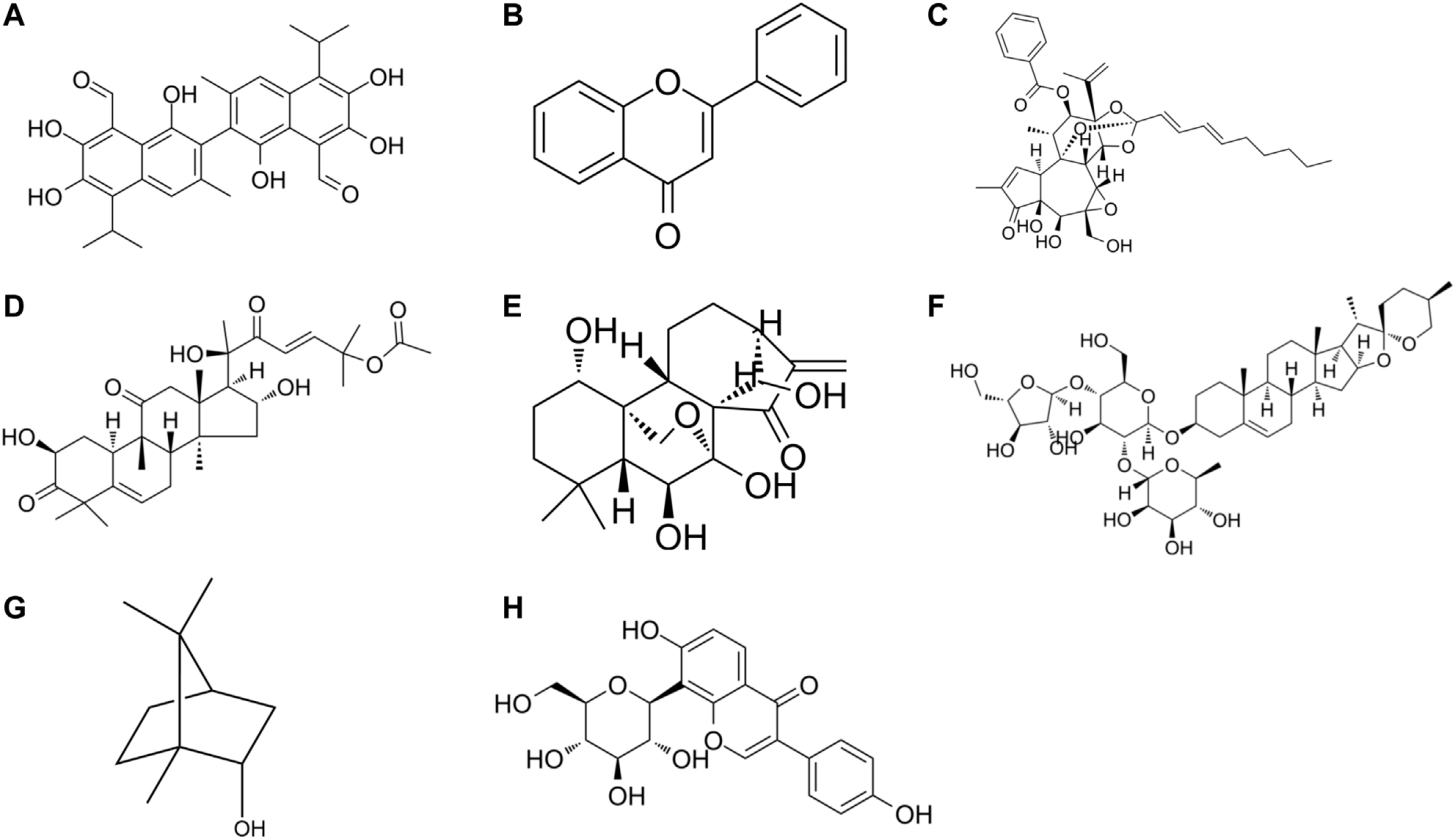
The chemical formula of drug monomers **(A)** Gossypol, **(B)** Flavone, **(C)** Yuanhuadine, **(D)** Cucurbitacin B, **(E)** Oridonin, **(F)** Polyphyllin I, **(G)** natural borneol, **(H)** Puerarin.

TKIs targeting EGFR are the standard treatment for NSCLC patients with EGFR mutations, and their application can significantly improve the quality of life of NSCLC patients with EGFR mutations. But almost all patients treated with targeted drugs eventually inevitably develop drug resistance after a period of treatment, affecting the prognosis and survival. It has been shown that resistance develops within 9–14 months in most patients with EGFR-mutated NSCLC treated with first- or second-generation EGFR-TKIs, such as gefitinib, erlotinib, and afatinib (Mok et al., 2009; Rosell et al., 2012; Tricker et al., 2015). Although third-generation EGFR-TKIs have therapeutic advantages for NSCLC patients with the T790M mutation, most patients will become resistant to these agents and will eventually lead to disease progression (Zhang et al., 2020b). Therefore, it is important to investigate the treatment after EGFR-TKIs resistance to solve the “neck clamping” problem of resistance. Herbs and active compounds have a non-negligible potential to overcome drug resistance and adverse effects of EGFR-TKIs (Figure 2 and Table 2).

**TABLE 2:** The potential role of herbs and active compounds in overcoming drug resistance and adverse effects of EGFR-TKIs.

Drug monomers/Traditional Chinese medicine compound	Anti-drug resistance	Drug monomers/Traditional Chinese medicine compound	Anti-adverse effects
Gossypol	YAP/TAZ	Puerarin	Blood hypercoagulability
	EGFR L858R/T790M		
Trifolium flavonoids	ERK pathways STAT3 pathways	Xiaofeng Powder	EGFR-TKIs-associated rash
	Bcl2 and Mcl-1		
Yuanhuadine	NNMT miRNA-449a	Pipaqingfeiyin	EGFR-TKIs-associated rash
Cucurbitacin B	PI3K/Akt/mTOR pathways	Zhiyang Pingfu Decoction	EGFR-TKIs-associated rash
Oridonin	EGFR/ERK/MMP12 CIP2A/PP2A/Akt pathways	Yangyin Zhixie recipe	EGFR-TKIs-associated diarrhea
Polyphyllin I	IL-6/STAT3 pathways	Honeysuckle	EGFR-TKIs-associated rash
Yiqi Chutan Decoction	apoptosis and autophagy	Stragalus, Ginseng, and Atractylodes macrocephala-based herbal formulae	3–4 serious side effects

### 4.1 Drug monomers

Gossypol is a polyphenol hydroxybisnaphthalene aldehyde compound that is mainly present in the roots, stems, leaves, and seeds of Malvaceae plant cotton. Combination therapy of gossypol and gefitinib can significantly reduce H1975 cell growth compared with gefitinib alone. Gossypol-treated H1975 cells are more sensitive to gefitinib, which can target YAP/TAZ and EGFR L858R/T790M to overcome EGFR-TKIs resistance (Xu et al., 2019).

Flavonoids are phenolics widely present in leaves, flowers, and fruits of plants, and have some potential in anti-tumor (Wang et al., 2021a). Some scholars have found that the combination of subtoxic doses of trifolium flavonoids and gefitinib can significantly inhibit the proliferation and induce apoptosis of human NSCLC gefitinib-resistant PC-9R cell lines, and enhance the chemosensitivity of gefitinib-resistant cells to gefitinib. The mechanism is related to the downregulation of ERK and STAT3 signaling pathways and the decrease of Bcl2 and Mcl-1 expression levels (Wu et al., 2020).

Nicotinamide N-methyltransferase (NNMT) is a metabolic enzyme associated with tumor-associated fibroblast (CAF) differentiation and tumor progression (Eckert et al., 2019), low levels of NNMT inhibited p-Akt and tumor development. While miR-449a expression induced phosphatase and tensin homolog (PTEN) and inhibited tumor growth. In EGFR-TKIs-resistant NSCLC cells, NNMT expression is upregulated, miR-449a is downregulated, and miR-449a expression level affects the sensitivity of NSCLC cells to gefitinib. Yuanhuadine, as an active ingredient in the traditional Chinese medicine Daphne genkwa, can regulate NNMT and miRNA-449a expression, thereby overcoming EGFR-TKIs resistance in NSCLC.

Cucurbitacin B (CuB) is a tetracyclic triterpenoid compound isolated from cucurbitaceae and other plants, which has been confirmed to have good anti-inflammatory and anticancer effects. A basic study (Yuan et al., 2022) found that CuB could inhibit TGF-β1-induced EMT and limit tumor cell migration and invasion in A549 cells. Moreover, CuB could inhibit EMT in gefitinib-resistant A549 cells through ROS and PI3K/Akt/mTOR pathways.

Oridonin (Ori) is a bioactive natural substance isolated from Rabdosia plants of the Labiatae family, which has various biological activities and pharmacological effects such as anti-tumor, anti-inflammatory, neuroprotective and antibacterial effects. Oridonin has been found (Xiao et al., 2016) to be an effective candidate for the treatment of gefitinib-resistant NSCLC by inhibiting EGFR/ERK/MMP-12 and CIP2A/PP2A/Akt signaling pathways, thereby inhibiting the proliferation, invasion, and migration of gefitinib-resistant NSCLC cells.

Polyphyllin I (PPI) is a natural compound isolated from the rhizomes of Paris polyphylla and has various pharmacological activities such as anti-tumor, anti-inflammatory, immunomodulatory, and antioxidant activities. PPIs have been found (Lou et al., 2017) to reverse EMT and decrease IL-6/STAT3 signaling pathways in erlotinib-resistant cells by restoring drug sensitivity in NSCLC acquired resistant cells. Moreover, erlotinib combined with PPIs effectively inhibited tumor growth in xenografts, providing a new clinical option to overcome EGFR-TKI resistance in NSCLC.

Blood hypercoagulability is a common concomitant symptom of NSCLC and is particularly likely to occur in patients with mid-to late-stage NSCLC. Blood hypercoagulation not only promotes the growth and metastasis of malignant tumors, but also promotes the formation of blood clots, which can lead to embolism of arteries and veins and vital organs. Puerarin is an isoflavone derivative with coronary dilation effect isolated from the Chinese medicine Pueraria lobata. It has antipyretic and sedative effects and is clinically useful for the treatment of coronary heart disease and hypertension. The efficacy of Puerarin combined with gefitinib was found to be significantly better than that of gefitinib treatment in the study. It was also significantly more effective than gefitinib treatment in improving the hypercoagulable state of the patient’s blood (Wu et al., 2022).

With the intensive development of drugs, the combination of nanotechnology and natural drugs is being used for cancer therapy. Natural chemical sensitizers natural borneol (NB) were formulated as oil-in-water nanoemulsions, and nanosized NB (nbnp) showed stronger targeted delivery and cytotoxicity than NB. nBNPs achieved stronger chemosensitization than NB and gefitinib by effectively regulating EGFR/EHD1-mediated apoptosis in A549 NSCLC cells. nBNPs and gefitinib’s synergistic effect not only enhanced the anti-cancer ability of gefitinib against NSCLC proliferation, but also avoided the severe dual toxicity *in vivo* (Yuan et al., 2020).

### 4.2 Traditional Chinese medicine compound

Yiqi Chutan Decoction (YQCT) is a prescription composed of eight herbs including American Ginseng (Xi Yang Shen), Bulbus Fritillariae Thunbergii (Zhe Bei Mu), and Radix Ranunculi Ternati (Mao Zhua Cao). It had confirmed its efficacy in inhibiting lung cancer tumor growth and reducing drug resistance (Wang et al., 2011; Zhang et al., 2016). Compared with gefitinib alone, gefitinib combined with YQCT treatment can significantly reduce H1975 cell viability, inhibit cell proliferation and DNA synthesis, and reduce gefitinib-induced NSCLC resistance by targeting apoptosis and autophagy (Zhang et al., 2020a).

Jinfukang decoction (JFKD) is a traditional Chinese medicine oral liquid, which is made from 12 kinds of traditional Chinese medicines including Astragalus membranaceus, Radix Ginseng, and Radix Asparagi. It was approved by the State Drug Administration in 1999 for the treatment of NSCLC. The combination of JFKD and gefitinib in gefitinib-resistant cells significantly enhanced the effect on tumor cells, PC-9/wt cells and PC-9/gef cells apoptosis rate can be as high as 49.7% and 40.6%. JFKD + gefitinib group inhibited the growth of gefitinib-resistant xenografts *in vivo* more than JFKD or gefitinib alone (*p* < 0.05) (Huang et al., 2021).

Some herbal compound formulas also have significant alleviating effects on EGFR-TKIs-associated AE. Xiaofeng Powder, Pipaqingfeiyin (Dong et al., 2016) and Zhiyang Pingfu Decoction (Wang et al., 2015) have good effects on EGFR-TKIs-associated rash and can improve patients’ quality of life. Yangyin Zhixie recipe can effectively relieve the systemic symptoms of EGFR-TKIs-associated diarrhea and improve the quality of life of patients with good safety (Wang, 2019).

### 4.3 Clinical studies

Some scholars have carried out a stratified prospective multicenter cohort study (Tang, 2019) to observe the efficacy of traditional Chinese medicine combined with EGFR-TKIs. A total of 153 patients with EGFR positive stage IIIb/IV NSCLC were enrolled and divided into the test group treated with EGFR-TKIs combined with traditional Chinese medicine and the control group treated with EGFR-TKIs alone. The results showed that the median PFS was 13.0 vs. 8.8 mth (*p* = 0.001) in the test and control groups, 11 vs. 8.5 months (*p* = 0.007) in the test and control groups in patients with exon 19 deletion mutations, and 14 vs. 9.5 months (*p* = 0.015) in the test and control groups in patients with exon 21 deletion mutations. DCR was 90.11% vs. 83.33% in the active and control groups (*p* = 0.219). Chinese medicine combined with EGFR-TKIs can prolong PFS and improve clinical prognosis in patients with advanced NSCLC.

A real-world study (Yang, 2020) to observe the synergistic effect of elemene, a traditional Chinese medicine extract, combined with first-generation EGFR-TKIs in the treatment of advanced lung adenocarcinoma. 113 patients with stage IIIB-IV lung adenocarcinoma were divided into elemene combined with EGFR-TKIs group, EGFR-TKIs alone group and conventional chemoradiotherapy group. The results showed that the mPFS of elemene + EGFR-TKIs group, EGFR-TKIs alone group and chemoradiotherapy group were 14.0 months, 10.0 months (*p* = 0.037) and 7.5 months (*p* = 0.002), respectively. The DCR in the elemene + EGFR-TKIs group was higher than that in the chemoradiotherapy group (59.4%) (*p* = 0.0008), and a higher disease control rate could be achieved with the combination therapy. In smoking patients, PFS was significantly longer in the elemene plus EGFR-TKIs group than in both the EGFR-TKIs group (*p* = 0.029) and the chemoradiotherapy group (*p* = 0.027). Compared with EGFR-TKIs group and chemoradiotherapy group, elemene + EGFR-TKIs group showed significant improvement in quality of life (*p* < 0.05). Moreover, the incidence of rash was significantly lower in the elemene + EGFR-TKIs group (*p* = 0.034). Therefore, elemene combined with EGFR-TKIs has a potential synergistic effect.

Some scholars have carried out a randomized controlled trial (Yang et al., 2018) to observe the clinical efficacy of Yiqi Yangyin Sanjie Decoction combined with EGFR-TKIs in the treatment of advanced NSCLC with secondary resistance to EGFR-TKIs. Patients with advanced NSCLC resistant to EGFR-TKIs were randomly divided into chemotherapy group (docetaxel or pemetrexed with or without cisplatin/carboplatin), EGFR-TKIs group and Yiqi Yangyin Sanjie Decoction + EGFR-TKIs group. The results showed that compared with EGFR-TKIs group and chemotherapy group, the DCR of Yiqi Yangyin Sanjie Decoction + EGFR-TKIs group was significantly prolonged (29.6% vs. 32.0% vs. 46.9%). The median PFS was 4.8 months and the median OS was 29.4 months in the Yiqi Yangyin Sanjie Decoction + EGFR-TKIs group, which was not statistically different from the EGFR-TKIs group (median PFS 3.9 months, median OS 26.1 months) and the chemotherapy group (median PFS 5.0 months, median OS 20.1 months), and the median OS was significantly prolonged (*p* < 0.05). Moreover, the clinical syndrome and quality of life of the combined treatment group were effectively improved. It can be seen that Yiqi Yangyin Sanjie Decoction combined with EGFR-TKIs can improve the prognosis of patients, and has good safety.

A randomized, double-blind, placebo-controlled prospective clinical study (Zhu et al., 2018) observed the efficacy and safety of Fuzheng Zhiai herbs combined with EGFR-TKIs targeted drugs in the treatment of advanced NSCLC. 60 patients with advanced NSCLC with EGFR gene mutation were randomly divided into two groups: The experimental group treated with Fuzheng Zhiai syndrome differentiation herbs + targeted therapy and the control group treated with placebo + targeted therapy. The results showed that the median PFS was 4.7 months longer in the test group than in the control group (15.20 ± 2.43 vs. 10.5 ± 1.7, *p* = 0.215). The median OS was 6.7 months longer in the test group than in the control group (30.2 ± 4.0 vs. 23.5 ± 2.6, *p* = 0.387). In addition, the improvement of TCM clinical syndrome of Qi deficiency type patients in the test group was significantly better than that in the control group (*p* = 0.001). The improvement of LSCC scale symptoms was significantly better than that in the control group (*p* = 0.001). There was no significant difference in the overall incidence of treatment-related adverse drug reactions between the two groups. Therefore, Fuzheng Zhiai Chinese herbal formula has a tendency to prolong PFS and OS in patients with advanced NSCLC treated with TKIs targeted therapy. It improved the symptoms and quality of life in patients, reduced the occurrence of serious adverse events.

Most patients treated with EGFR-TKIs develop skin toxicity, which can affect quality of life and lead to discontinuation of cancer treatment. Honeysuckle is a traditional herb historically used in East Asia for the treatment of skin rashes and has a proven efficacy and safety profile. 139 patients with tumors treated with EGFR-TKIs were recruited to apply honeysuckle treatment. Honeysuckle was effective in reducing the incidence and severity of EGFR-TKIs-induced acne-like rash by 10%–21% and faster recovery of pruritus compared with conventional treatment (minocycline) (Liu et al., 2022).

In a clinical cohort study, 30 patients were treated with EGFR-TKIs alone and 61 patients were treated with EGFR-TKIs + Chinese herbs (Astragalus, Ginseng, and Atractylodes macrocephala-based herbal formulae) were shown. The results showed that the median survival was prolonged by 3.4 months (12.3 vs. 8.9) in the EGFR-TKIs combined with herbal medicine treatment group. The incidence of grade 3–4 serious side effects, such as rash, diarrhea, liver damage, oral ulcers, and paronychia, was reduced by 15.19% (26.67% vs. 11.48%). Chinese medicine combined with EGFR-TKIs has a tendency to enhance efficacy, prolong survival and reduce drug side effects (Wang et al., 2021b).

## 5 Conclusion and perspectives

Compared with traditional chemotherapy, EGFR-TKIs-targeted therapy shows advantages not only in efficacy, tolerability, adverse events and survival benefit, but equally importantly, quality of life. It has become one of the effective treatments for advanced NSCLC. Although targeted therapies have improved the prognosis of patients with NSCLC, the effects of these agents are only temporary or partially effective. Targeted drug resistance remains the most significant problem hindering targeted therapies in NSCLC today. The development and application of second- and third-generation EGFR-TKIs drugs still cannot fundamentally overcome drug resistance, which is due to the complex network system formed between genes and signaling pathways regulating tumor growth. The inhibition of a single target by drugs is often replaced by activation of bypass signaling pathways, leading to continued tumor growth, which ultimately does not achieve satisfactory therapeutic effects. The advantage of natural drugs lies in the overall regulation of multiple targets, which has the advantage of potentially improving or reversing drug resistance, promoting body balance and stability by regulating the internal environment of cancer patients, and playing a synergistic role with targeted drugs. The holistic regulation of natural herbs combined with precise targeted therapy with EGFR-TKIs has macroscopic and microscopic complementary effects, and has the potential to become an innovative model for lung cancer treatment.

Therefore, recognizing the complex molecular alterations underlying the development of resistance to targeted therapies is necessary to understand the mode of tumor cells survival during treatment, clinical-basic translation, design drug combinations and design therapeutic strategies to prevent drug resistance. Targeted combination chemotherapy, anti-angiogenic therapy, immunotherapy and natural drug combination therapy have achieved initial results, but further exploration and research are needed for the details in screening of the population with advantageous benefits of combination regimens, optimal combination drug regimens, management of toxic side effects and implementation of individualized diagnosis and treatment regimens. Lots of preclinical studies have also explained various possible resistance mechanisms, providing ideas for the development of novel drugs. Efforts to explore various therapeutic sequences, combinations and to conduct development of novel targeted agents for better efficacy are the goal of our future development.

With the continuous update and application of targeted drugs related to NSCLC, the prevention and treatment of related adverse events have received increasing attention. The effectiveness of natural medicines in mitigating and ameliorating their toxic side effects is worthy affirming. Integrative medicine research has further reduced the impact of adverse events on patients, and bring hopes for good prognosis. There are still two issues that need to be solved: 1) Traditional Chinese medicine (TCM) has no standard treatment regimen for various forms of adverse events occurring during the use of EGFR-TKIs; 2) Although TCM have certain efficacy on EGFR-TKIs-related adverse events, large randomized controlled trials and complete expert consensus are still needed to increase their persuasion. At present, research on the combined application of natural medicines with EGFR-TKIs is still in its infancy, and further in-depth exploration of its mechanism of action is needed to continuously improve the effect of targeted lung cancer therapy. In the future research, we will conduct further research on the potential of herbs and active compounds to overcome the drug resistance and side effects of EGFR-TKIs through clinical and animal experiments.
